# Lipid Peroxides Mediated Ferroptosis in Electromagnetic Pulse-Induced Hippocampal Neuronal Damage via Inhibition of GSH/GPX4 Axis

**DOI:** 10.3390/ijms23169277

**Published:** 2022-08-17

**Authors:** Yunfei Lai, Ji Dong, You Wu, Li Zhao, Hui Wang, Jing Zhang, Binwei Yao, Xinping Xu, Yong Zou, Haixia Zhao, Hanlin Yue, Yiwei Song, Haoyu Wang, Ruiyun Peng

**Affiliations:** Beijing Institute of Radiation Medicine, Beijing 100850, China

**Keywords:** electromagnetic pulse radiation, hippocampal neuronal damage, ferroptosis, lipid peroxides, glutathione peroxidase 4

## Abstract

Electromagnetic pulse (EMP) radiation was reported to be harmful to hippocampal neurons. However, the mechanism underlying EMP-induced neuronal damage remains unclear. In this paper, for the first time, we attempted to investigate the involvement of ferroptosis in EMP-induced neuronal damage and its underlying mechanism. In vivo studies were conducted with a rat model to examine the association of ferroptosis and EMP-induced hippocampal neuronal damage. Moreover, in vitro studies were conducted with HT22 neurons to investigate the underlying mechanism of EMP-induced neuronal ferroptosis. In vivo results showed that EMP could induce learning and memory impairment of rats, ferroptotic morphological damages to mitochondria, accumulation of malonaldehyde (MDA) and iron, overexpression of prostaglandin-endoperoxide synthase 2 (PTGS2) mRNA, and downregulation of GPX4 protein in rat hippocampus. In vitro results showed that EMP could induce neuronal death, MDA accumulation, iron overload, PTGS2 overexpression, and GPX4 downregulation in HT22 neurons. These adverse effects could be reversed by either lipid peroxides scavenger ferrostatin-1 or overexpression of GPX4. These results suggest that EMP radiation can induce ferroptosis in hippocampal neurons via a vicious cycle of lipid peroxides accumulation and GSH/GPX4 axis downregulation. Lipid peroxides and the GSH/GPX4 axis provide potential effective intervention targets to EMP-induced hippocampal neuronal damage.

## 1. Introduction

The World Health Organization (WHO) has listed electromagnetic radiation as one of the most common and fastest growing sources of environmental pollution [1]. Electromagnetic pulse (EMP) is a specific form of electromagnetic wave with a high voltage, a short pulse duration, and a broad bandwidth [2]. EMP radiation mainly occurs in certain occupational conditions, such as pulse power technology labs and military environments [2]. There is increasing evidence that EMP radiation can endanger human health, especially the central nervous system (CNS) [3,4,5,6,7,8,9]. Previous studies suggested that the damage to learning and memory caused by EMP radiation might be associated with the activation of oxidative stress in neurons [3,5,6]. Additionally, EMP radiation could lead to neuronal cell death, such as apoptosis, which would further cause CNS damage [4,10]. However, the mechanism underlying EMP-induced hippocampal neuronal damage is still not fully understood.

Ferroptosis is a form of regulated cell death (RCD) characterized by iron-dependent accumulation of lipid peroxides. Ferroptosis is morphologically, biochemically, and genetically distinct from other forms of RCD, such as apoptosis, necrosis, and autophagy [11,12,13]. The unique morphological features of ferroptosis are mitochondrial abnormalities, including swollen or condensed mitochondria, outer membrane rupture, and reduced numbers or lack of cristae at the ultrastructural level [11,14]. Ferroptosis is biochemically characterized by the requirement for iron and the accumulation of lipid peroxides. Moreover, one of the most important genetic features of ferroptosis is the upregulation of prostaglandin-endoperoxide synthase 2 (PTGS2) mRNA [13]. It has been suggested that ferroptosis exerts important effects on the pathological processes of various neurological diseases [15,16,17,18]. However, whether ferroptosis is involved in EMP-induced hippocampal neuronal damage remains unknown.

Ferroptosis mainly arises from maladaptation of three metabolic pathways: lipid peroxidation accumulation, glutathione inhibition, and iron overload [19,20]. A fatal intracellular accumulation of lipid peroxidation and the disorders of its product lipid hydroperoxides (LOOHs) are essential to ferroptosis [21]. It has been demonstrated that EMP can induce oxidative damage to the cellular membrane system and cause lipid peroxidation [22], indicating that accumulation of lipid peroxides may be a vital factor in EMP-induced cytotoxicity. Moreover, as a key factor in glutathione metabolic pathways, glutathione peroxidase 4 (GPX4) has been proven to be a central regulator of ferroptosis [23]. It has been demonstrated that both direct and indirect suppression of GPX4 could induce ferroptosis [12]. Glutathione peroxidases (GPXs) are a group of enzymes that could catalyze the reduction in hydrogen peroxide and organic hydroperoxides to water or the corresponding alcohols, using glutathione (GSH) as an essential cofactor [24]. Unlike other GPX enzymes, GPX4 can catalyze the reduction in lipid peroxides [24], which makes the GSH/GPX4 axis the predominant system detoxifying phospholipid hydroperoxides (PLOOH) [20]. A previous study reported that EMP radiation caused associative learning deficits by increasing the levels of lipid peroxides, depletion of GSH, and decreasing the levels of GPXs in mouse brains [4], implying a possible inhibition of the GSH/GPX4 axis in EMP-induced neuronal injury and the corresponding functional impairments.

The main purpose of this work was to shed light on the role of ferroptosis in EMP-induced hippocampal neuronal damage and the related underlying mechanism. To achieve these goals, studies were conducted with both animal and cell models. First, in vivo studies were conducted with a rat model to examine the association of ferroptosis and EMP-induced hippocampal neuronal damage. Then, in vitro studies were conducted with HT22 neurons pretreated with a ferroptosis inhibitor and lipid peroxides scavenger, ferrostatin-1 (Fer-1), to further investigate the involvement of ferroptosis in EMP-induced hippocampal neuronal death. Specifically, the underlying mechanism of EMP-induced neuronal ferroptosis was also investigated using HT22 neurons with GPX4 overexpression. This work not only laid a foundation for revealing the mechanism underlying EMP-induced hippocampal neuronal damage but also showed the potential for developing therapeutic approaches based on the inhibition of ferroptosis.

## 2. Results

### 2.1. The Increase in Temperature during EMP Exposure Was Less Than 1 °C

**Figure 1:**
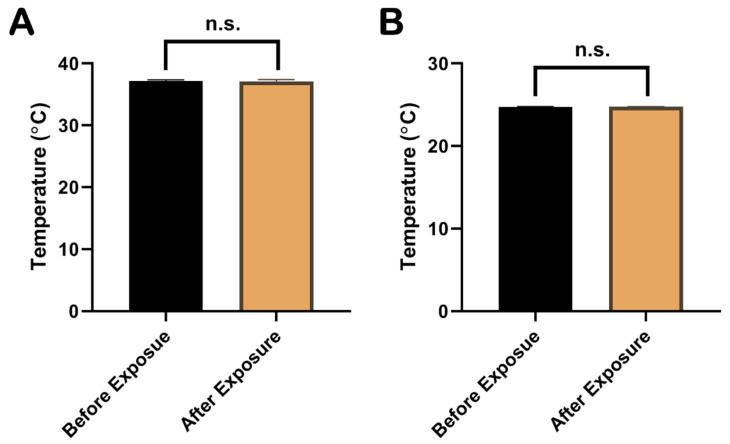
Comparison of temperatures in rat rectum and cell culture medium before and immediately after EMP exposure. (**A**) showed the comparison of rectum temperatures in rat before and immediately after EMP exposure (n = 3). (**B**) showed the comparison of temperatures in cell culture medium before and immediately after EMP exposure (n = 3). Significance was calculated using paired *t*-test; n.s., not significant. Abbreviations: EMP, electromagnetic pulse.

The temperature of the rat rectum and the cell culture medium were measured with a fiber thermometer during the whole EMP exposure procedure. The results showed that the temperature changes in the rat rectum (Figure 1A) and the cell culture medium (Figure 1B) before and immediately after the EMP exposure were <±1 °C and <±0.5 °C, respectively. As shown in Figure 1, there were no significant changes in temperatures of rat rectum and the cell culture medium (*p* > 0.05 and *p* > 0.05, respectively).

### 2.2. EMP Exposure Could Induce Spatial Learning and Memory Impairment in Rats

**Figure 2:**
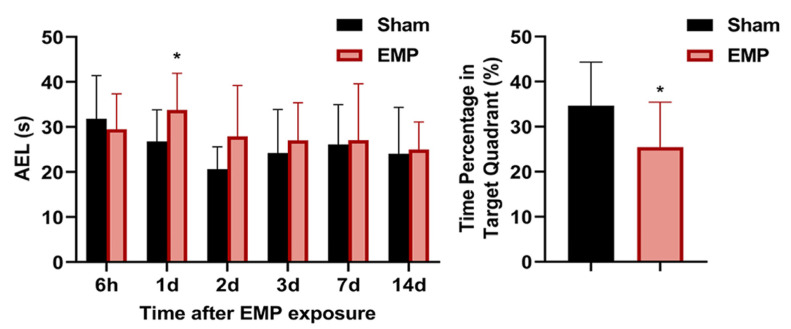
EMP exposure could induce spatial learning and memory impairment in rats. Comparison of AELs at 6 h, 1 d, 2 d, 3 d, 7 d, and 14 d after the EMP exposure (Sham: n = 10; EMP: n = 11) and the time percentages in the target quadrant at 4 d after the EMP exposure (Sham: n = 10; EMP: n = 11). Significance was calculated using two-tailed unpaired Student’s *t*-test; * *p* < 0.05 compared with the sham group. Abbreviations: AEL, the average escaping latency; EMP, electromagnetic pulse.

The MWM test was performed at 6 h, 1 d, 2 d, 3 d, 4 d, 7 d, and 14 d after the EMP exposure. In the navigation tests, the AELs of the rats in the EMP group were significantly prolonged at 1 d compared with those in the sham group (*p* < 0.05, Figure 2). At 4 d after the EMP exposure, the percent of time spent in the target quadrant of the EMP group was significantly shorter than that of the sham group (*p* < 0.05, Figure 2). Taken together, our findings suggested that EMP exposure significantly decreased the spatial learning and memory abilities in rats.

### 2.3. EMP-Induced Neuronal Injury in Rat Hippocampus Was Associated with Ferroptosis

**Figure 3:**
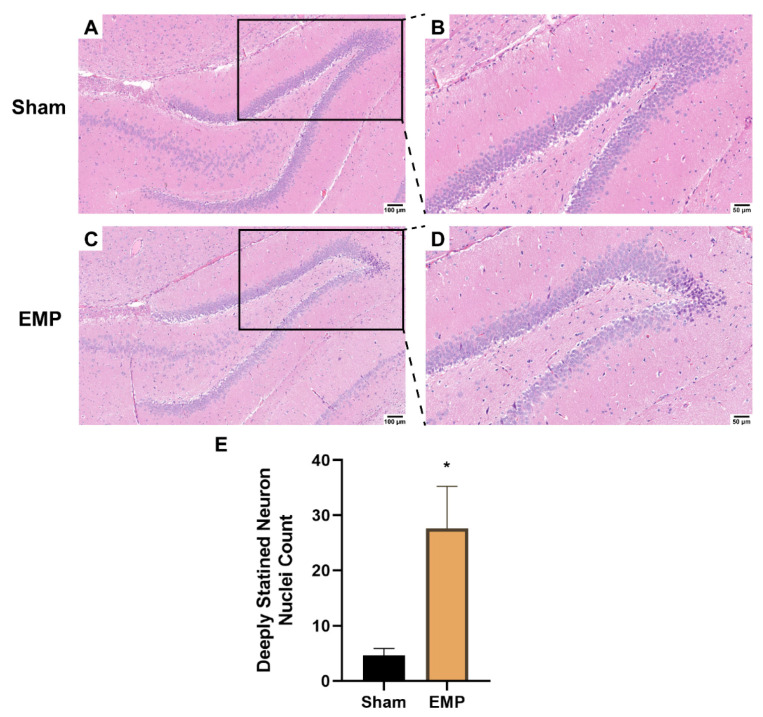
EMP exposure could induce microstructure damage to the hippocampus in rats. (**A**,**C**) showed the representative images of brain sections, including hippocampus, stained with HE obtained from the sham and EMP groups after the EMP exposure (n = 5 for each group; scale bar = 100 μm). (**B**,**D**) were enlargements of regions indicated with black rectangles in (**A**,**C**), respectively (scale bar = 50 μm). (**E**) Count of deeply stained neuron nuclei was measured in rat hippocampus after the EMP exposure (n = 5 for each group). Significance was calculated using two-tailed unpaired Student’s *t*-test; * *p* < 0.05 compared with the sham group. Abbreviations: EMP, electromagnetic pulse; HE, hematoxylin–eosin.

**Figure 4:**
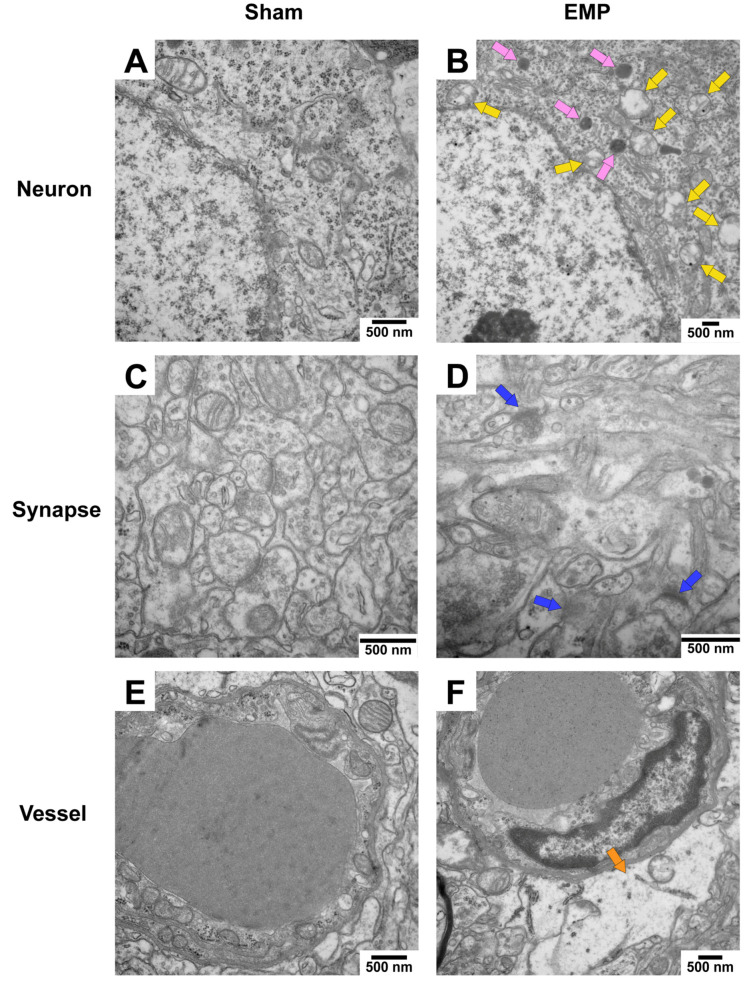
EMP exposure could induce ultrastructure damage to the hippocampus in rats. (**A**,**C**,**E**) showed that no obvious morphological abnormalities of hippocampal neurons, synapses, and vessels were observed in the sham group. (**B**,**D**,**F**) showed the damaged ultrastructures of hippocampal neurons, synapses, and vessels in the EMP group. The representative TEM images showing neurons (top row), synapses (middle row), and vessels (bottom row) in hippocampus obtained from the sham and EMP groups after the EMP exposure (n = 3 for each group; scale bar = 500 μm). Yellow arrows indicated the swollen mitochondria, pink arrows indicated the secondary lysosome, blue arrows indicated the blurred synaptic gaps, and orange arrows indicated the dilation of perivascular space. Abbreviations: EMP, electromagnetic pulse; TEM, transmission electron microscopy.

To explore the pathological damage to the hippocampus, histological examinations of the hippocampus were carried out after the EMP exposure. First, the microstructures of the hippocampus were observed using optical microscopy. The number of deeply stained neuron nuclei around the dentate gyrus (DG) area in rat hippocampus in the EMP group was significantly higher than in the sham group (*p* < 0.05, Figure 3A–E), which indicated neuronal degeneration in this region. Then, the ultrastructures of the hippocampus were also examined by TEM. As shown in Figure 4A,C,E, in the sham group, no obvious morphological abnormalities were observed in the nucleus and mitochondria of hippocampal neurons, synapses, or vessels. Compared with those of the sham group, the hippocampal neurons of the EMP group exhibited specific morphological features of ferroptosis, including mitochondrial abnormalities, such as swelling, increased membrane density, and reduced numbers or lack of cristae (Figure 4B). Moreover, the increased numbers of secondary lysosomes in the hippocampal neurons, blurred synaptic clefts, and obvious dilation of perivascular spaces were also observed after the EMP exposure (Figure 4B,D,F, respectively). In summary, these results suggested that both the micro-and ultra-structures of the hippocampus were damaged after the EMP exposure.

**Figure 5:**
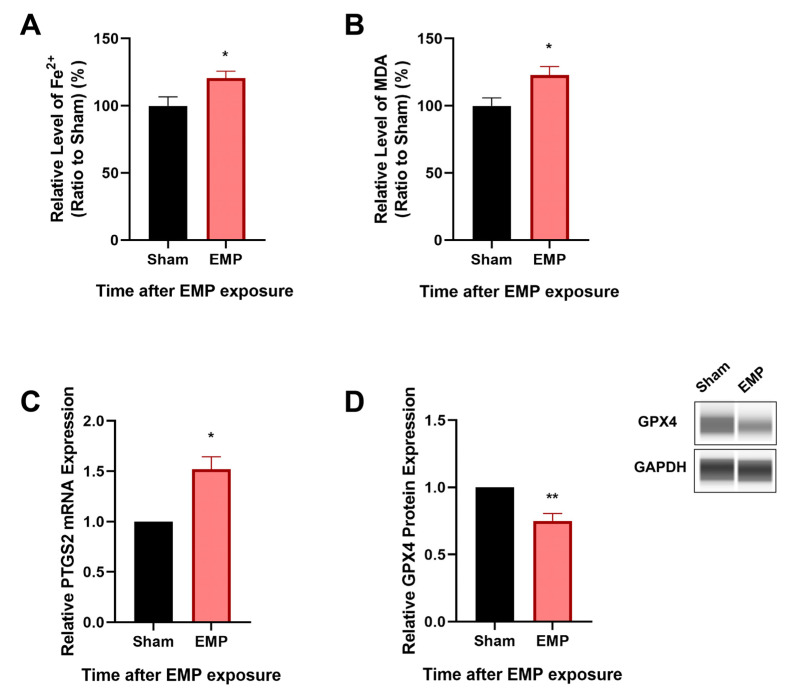
EMP exposure-induced neuronal injury was associated with ferroptosis in rat hippocampus. (**A**) Fe^2+^ level (Sham: n = 12; EMP: n = 15), (**B**) MDA level (Sham: n = 10; EMP: n = 9), (**C**) PTGS2 mRNA expression (Sham: n = 4; EMP: n = 4), and (**D**) GPX4 protein expression (Sham: n = 5; EMP: n = 5) were measured in the rat hippocampus after the EMP exposure. PTGS2 mRNA levels in (**C**) were normalized to GAPDH mRNA and were expressed relative to the respective mean sham value. Significance was calculated using two-tailed unpaired Student’s *t*-test; * *p* < 0.05 and ** *p* < 0.01 compared with the sham group. Abbreviations: EMP, electromagnetic pulse; GAPDH, glyceraldehyde 3-phosphate dehydrogenase; GPX4, glutathione peroxidase 4; MDA, malonaldehyde; PTGS2, prostaglandin-endoperoxide synthase 2.

To investigate the association of ferroptosis and the neuronal injury in rat hippocampus caused by EMP exposure, the levels of putative biomarkers of ferroptosis [13], including Fe^2+^, MDA, and PTGS2 mRNA, were measured after exposure. Moreover, the expression level of GPX4, a cornerstone of the antioxidant defense in ferroptosis, was also investigated. As shown in Figure 5A–C, we observed that the levels of Fe^2+^, MDA, and the mRNA expression of PTGS2 significantly increased (*p* < 0.05, *p* < 0.05, and *p* < 0.05, respectively) in rat hippocampus after the EMP exposure. Meanwhile, the expression of GPX4 significantly decreased (*p* < 0.01, Figure 5D). These results implied that ferroptosis might participate in EMP-induced hippocampal neuron injury along with the downregulation of GPX4 expression.

### 2.4. EMP-Induced Cell Death in HT22 Neurons Was Associated with Ferroptosis

**Figure 6:**
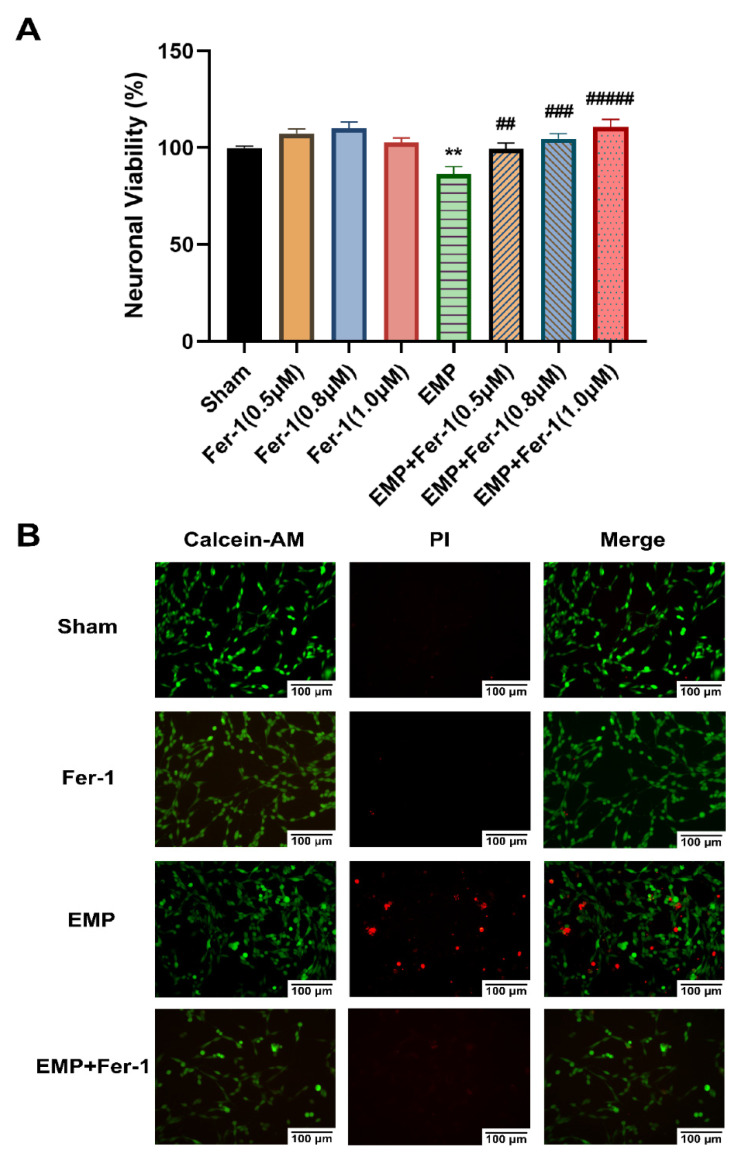
EMP-induced cell death in HT22 neurons could be alleviated by Fer-1. HT22 neurons were pre-treated with or without Fer-1 for 18 h and subsequently experienced EMP or sham exposures. (**A**) Neuronal viability of HT22 cells (sham: n = 5; 0.5 μM Fer-1: n = 6; 0.8 μM Fer-1: n = 7; 1.0 μM Fer-1: n = 6; EMP: n = 5; EMP + 0.5 μM Fer-1: n = 7; EMP + 0.8 μM Fer-1: n = 7; EMP + 1.0 μM Fer-1: n = 7) was measured after the EMP exposure. (**B**) The representative fluorescence images of the living and dead cells pre-treated with or without Fer-1 (1 μM) for 18 h were identified by Calcein AM/PI staining in the sham group (first row), the Fer-1 group (second row), the EMP group (third row), and the EMP+Fer-1 group (last row) after the EMP exposure (scale bar = 100 μm). Green fluorescent cells labeled with Calcein-AM indicated live cells; red fluorescent cells labeled with PI indicated dead cells. Significance was calculated using one-way ANOVA followed by LSD post hoc test; ** *p* < 0.01 compared with the sham group; ^##^ *p* < 0.01, ^###^ *p* < 0.001, and ^#####^ *p* < 0.00001 compared with the EMP group. Abbreviations: EMP, electromagnetic pulse; Fer-1, ferrostatin-1; Calcein-AM, Calcein acetoxymethyl ester; PI, propidium iodide.

To further investigate the effects of EMP on the hippocampal neurons, a CCK-8 assay and a Calcein-AM/PI double staining kit were used, respectively, to assess cell viability and cell death of an immortalized mouse hippocampal neuron line, HT22 neurons, after the EMP exposure. We observed that EMP exposure caused a significantly decreased cell viability (*p* < 0.01, Figure 6A) and an increased number of PI-stained dead cells (Figure 6B).

**Figure 7:**
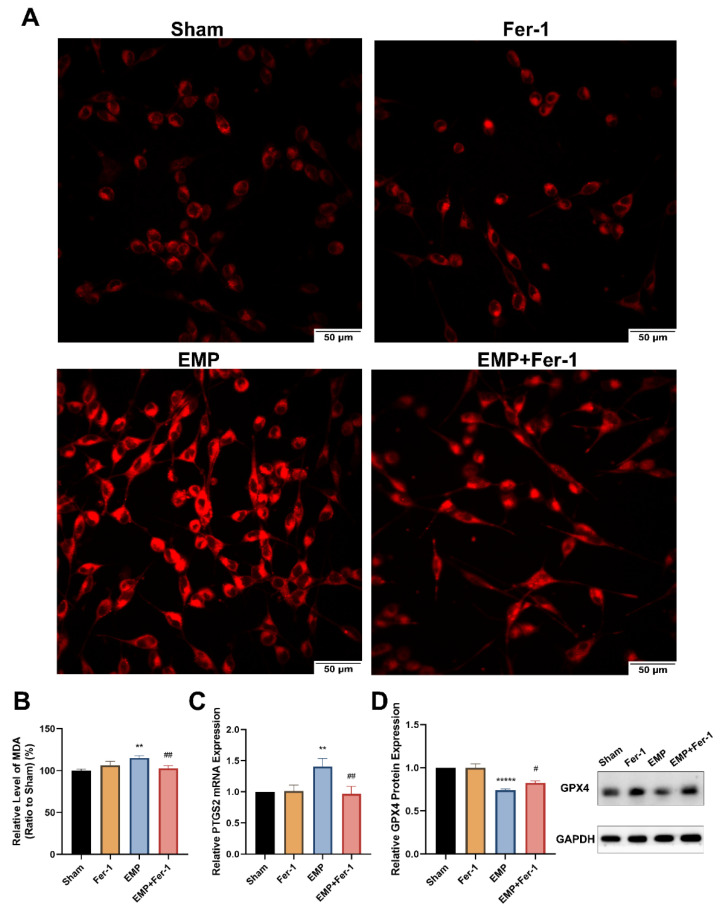
EMP-induced ferroptotic cell death in HT22 neurons could be alleviated by Fer-1. HT22 neurons were pre-treated with or without Fer-1 for 18 h and subsequently experienced EMP or sham exposures. (**A**) The representative fluorescence images of intracellular iron level pre-treated with or without Fer-1 (1 μM) for 18 h were measured in the sham group (top left), the Fer-1 group (top right), the EMP group (bottom left), and the EMP+Fer-1 group (bottom right) after the EMP exposure (scale bar = 50 μm). Red fluorescent cells labeled with FerroOrange indicated intracellular iron. (**B**) MDA level (Sham: n = 6; Fer-1: n = 5; EMP: n = 6; EMP+Fer-1: n = 6), (**C**) PTGS2 mRNA expression (Sham: n = 5; Fer-1: n = 4; EMP: n = 5; EMP+Fer-1: n = 4), and (**D**) GPX4 protein expression (n = 5) were measured after the EMP exposure. mRNA levels of PTGS2 were normalized to GAPDH mRNA and were expressed relative to the respective mean sham value. Significance was calculated using one-way ANOVA followed by LSD post hoc test; ** *p* < 0.01 and ***** *p* < 0.00001 compared with the sham group; ^#^ *p* < 0.05 and ^##^ *p* < 0.01 compared with the EMP group. Abbreviations: EMP, electromagnetic pulse; Fer-1, ferrostatin-1; GAPDH, glyceraldehyde 3-phosphate dehydrogenase; GPX4, glutathione peroxidase 4; MDA, malonaldehyde; PTGS2, prostaglandin-endoperoxide synthase 2.

To determine whether EMP-induced cell death in HT22 neurons was associated with ferroptosis, the putative biomarkers of ferroptosis were measured after the EMP exposure. We observed that EMP exposure could elevate the levels of iron (Figure 7A), MDA (*p* < 0.01, Figure 7B), and PTGS2 mRNA expression (*p* < 0.01, Figure 7C) in HT22 neurons after the EMP exposure. As shown in Figure 7D, EMP exposure significantly decreased the protein expression of GPX4 (*p* < 0.00001). Taken together, these results demonstrated that EMP-induced cell death in hippocampal neurons was associated with ferroptosis.

### 2.5. EMP-Induced Ferroptotic Cell Death in HT22 Neurons Could Be Alleviated by Fer-1

The effects of the ferroptosis inhibitor Fer-1 on the neuronal death caused by the EMP exposure were also examined. Notably, at various concentrations (0.5 μM, 0.8 μM, and 1.0 μM), Fer-1 elevated the viability of HT22 neurons in the EMP+Fer-1 group compared with that in the EMP group (EMP + 0.5 μM Fer-1: *p* < 0.01; EMP + 0.8 μM Fer-1: *p* < 0.001; EMP + 1.0 μM Fer-1: *p* < 0.00001, Figure 6A). In addition, cell viability increased at the increasing concentration of Fer-1 in the range of 0.5 μM–1.0 μM. Moreover, the number of PI-stained dead cells also decreased in the EMP+Fer-1 group compared with that in the EMP group (Figure 6B). These results indicated that the specific ferroptosis inhibitor Fer-1 treatment could protect HT22 neurons from EMP-induced cell death in a concentration-dependent manner.

Interestingly, Fer-1 treatment could markedly reverse the changes of putative biomarkers of ferroptosis caused by EMP exposure. Compared to the EMP groups, the level of MDA (*p* < 0.01, Figure 7B) and PTGS2 mRNA expression (*p* < 0.01, Figure 7C) in the EMP+Fer-1 groups decreased significantly. Moreover, Fer-1 treatment reversed the excessive Fe^2+^ level (Figure 7A). In addition, the protein expression levels of GPX4 significantly increased in the EMP+Fer-1 groups (*p* < 0.05, Figure 7D). These results indicated that ferroptosis was involved in EMP-induced hippocampal neuronal death.

### 2.6. EMP Exposure Could Affect the Antioxidant System in HT22 Neurons

**Figure 8:**
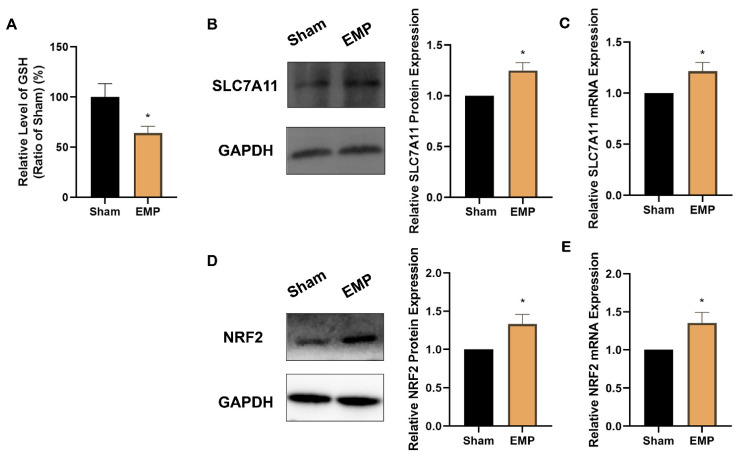
EMP exposure could affect the antioxidant system in HT22 neurons. (**A**) GSH level (Sham: n = 4; EMP: n = 6), (**B**) SLC7A11 protein expression (n = 5), (**C**) SLC7A11 mRNA expression (n = 5), (**D**) NRF2 protein expression (n = 6), and (**E**) NRF2 mRNA expression (n = 6) were measured after the EMP exposure. mRNA levels in (**C**,**E**) were normalized to GAPDH mRNA and were expressed relative to the respective mean sham value. Significance was calculated using two-tailed unpaired Student’s *t*-test; * *p* < 0.05, compared with the sham group. Abbreviations: EMP, electromagnetic pulse; GAPDH, glyceraldehyde 3-phosphate dehydrogenase; GSH, glutathione; NRF2, nuclear factor E2-related factor 2; SLC7A11, solute carrier family 7 member 11.

To investigate the role of key antioxidant factors in EMP-induced ferroptotic neuronal death, such as GPX4, GSH, NRF2, and SLC7A11, we examined their levels after the EMP exposure. The levels of protein expression of GPX4 (*p* < 0.00001, Figure 7D) and GSH (*p* < 0.05, Figure 8A) in HT22 neurons significantly decreased after the EMP exposure. Moreover, the mRNA and protein expression levels of SLC7A11 and NRF2 significantly increased (*p* < 0.05, *p* < 0.05, *p* < 0.05, and *p* < 0.05, respectively) after the EMP exposure (Figure 8B–E). These results implied that EMP exposure inhibited the GSH/GPX4 axis, although the NRF2/SLC7A11 pathway was activated as a compensatory response.

### 2.7. GPX4 Alleviated EMP-Induced Ferroptotic Neuronal Death in HT22 Neurons by Inhibiting Lipid Peroxides Accumulation

**Figure 9:**
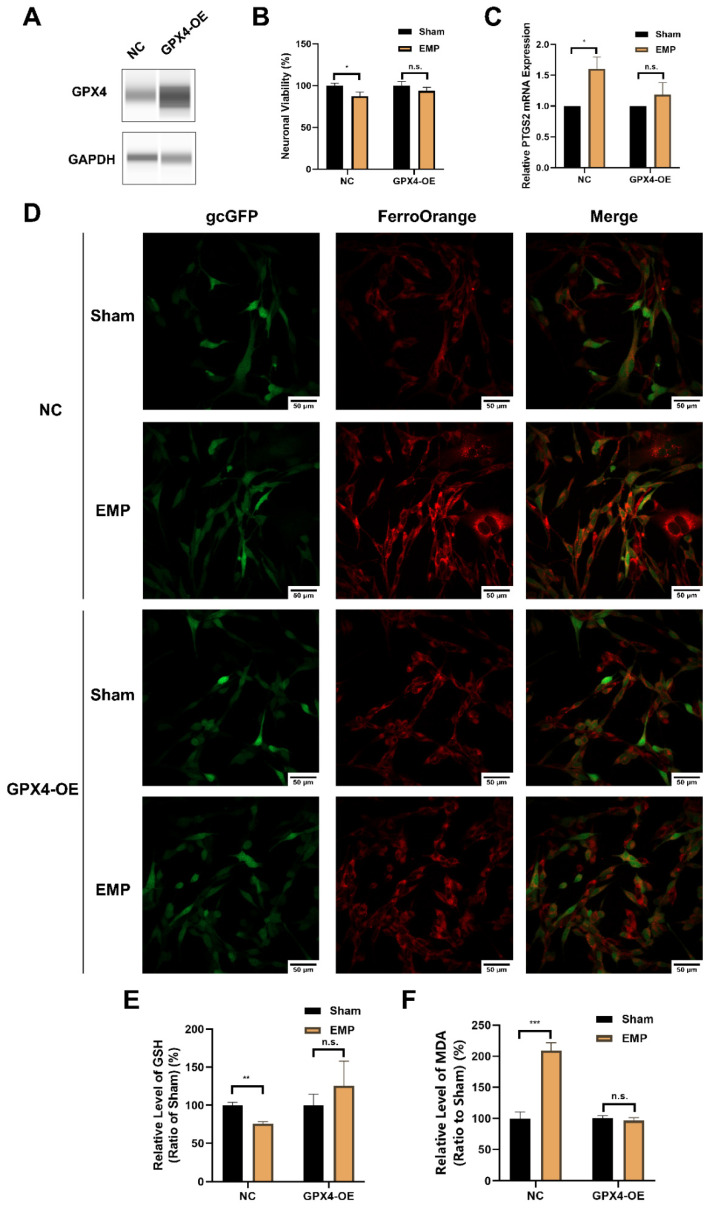
GPX4 alleviated EMP-induced ferroptotic neuronal death in HT22 neurons by inhibiting lipid peroxides accumulation. (**A**) HT22 neurons were transfected with GPX4-OE lentivirus or control lentivirus. GPX4 expressions were verified by Western blot; (**B**) Neuronal viability (n = 5), (**C**) PTGS2 mRNA expression (NC: n = 5; NC+EMP: n = 5; GPX4-OE: n = 6; GPX4-OE+EMP: n = 6), (**D**) The representative fluorescence images of intracellular iron level (scale bar = 50 μm; green, HT22 neurons transfected with GPX4-OE lentivirus or control lentivirus; red, intracellular iron), (**E**) GSH level (n = 5), (**F**) MDA level (NC: n = 6; NC+EMP: n = 6; GPX4-OE: n = 3; GPX4-OE+EMP: n = 3) were measured in the GPX4-OE or NC HT22 neurons after the EMP exposure. PTGS2 mRNA levels in (**C**) were normalized to GAPDH mRNA and were expressed relative to the respective mean sham value. Significance was calculated using two-tailed unpaired Student’s *t*-test; * *p* < 0.05, ** *p* < 0.01, *** *p* < 0.001 compared with the sham group; n.s., not significant. Abbreviations: EMP, electromagnetic pulse; GAPDH, glyceraldehyde 3-phosphate dehydrogenase; gcGFP, gc green fluorescent protein; GPX4-OE, glutathione peroxidase 4 overexpression; GSH, glutathione; MDA, malonaldehyde; NC, negative control; PTGS2, prostaglandin-endoperoxide synthase 2.

To elucidate the underlying mechanism of the GSH/GPX4 axis in EMP-induced ferroptotic neuronal death, HT22 neurons were transfected with a GPX4-overexpressing lentivirus. The overexpression of GPX4 was confirmed by Western blot (Figure 9A). As shown in Figure 9B–D, the overexpression of GPX4 reversed EMP-induced cell viability decrement, PTGS2 mRNA upregulation, and iron overload in HT22 neurons. Meanwhile, EMP-induced downregulation of GSH was alleviated by GPX4-OE HT22 neurons (Figure 9E). Moreover, the overexpression of GPX4 almost completely prevented EMP-induced MDA production (Figure 9F). Taken together, these findings suggested that activation of the GSH/GPX4 axis alleviated EMP-induced ferroptosis by inhibiting the accumulation of lipid peroxides.

## 3. Discussion

Electromagnetic radiation is one of the major sources of environmental pollution in the modern society. EMP, as a specific type of electromagnetic radiation, is also a potential health hazard to humans. Previous studies demonstrated that EMP could induce the hippocampal neuronal damage and impairment of spatial learning and memory [3,4,5,6,7,8,9]. Although numerous efforts have been made to elucidate the process by which EMP induces hippocampal neuronal injury, the mechanism is still unclear. In this paper, for the first time, we demonstrated the involvement of ferroptosis in the hippocampal neuronal damage and the spatial learning and memory impairment induced by EMP exposure. Moreover, the effects of lipid peroxides and the GSH/GPX4 axis on EMP-induced neuronal ferroptosis were also elucidated.

Ferroptosis, an evolutionarily conserved program, is an iron-dependent form of regulated cell death characterized by accumulation of lipid peroxides [11,13]. It was suggested that ferroptosis could be involved in the pathological processes of various neurological diseases, such as Alzheimer’s disease, Parkinson’s disease, traumatic brain injury, and stroke [15,16,17,18,25]. However, the role of ferroptosis in EMP-induced hippocampal neuronal damage and the related learning and memory impairment is still a virgin field. In this study, the involvement of ferroptosis in EMP-induced hippocampal neuronal damage was examined by both in vivo and in vitro studies. In vivo studies showed that EMP exposure could induce spatial learning and memory impairment in rats. Meanwhile, the morphological damage in the rat hippocampal neurons was observed after the EMP exposure. It was reported that the unique morphological features of ferroptotic cells included swelling mitochondria or shrunken mitochondria with increased membrane density and reduced numbers or lack of mitochondrial cristae [11,13,26]. The mitochondrial abnormalities observed in the present study were consistent with the morphological features of ferroptosis described in the previous literature. Moreover, the hallmarks of ferroptosis [11,12,13,23,27], such as iron overload, lipid peroxide accumulation, and increased PTGS2 mRNA expression, were also observed in rat hippocampus after the EMP exposure. In vitro studies demonstrated that EMP exposure could decrease cell viability, increase cell death, induce iron overload, MDA elevation, and upregulate the expression of PTGS2 in HT22 neurons. More importantly, all of these adverse effects could be reversed by Fer-1, the selective inhibitor of ferroptosis. Taken together, these in vivo and in vitro results provided convincing evidence that neuronal ferroptosis was involved in EMP-induced hippocampal neuronal damage, and ferroptosis was a key pathogenic factor in the corresponding spatial learning and memory impairment.

The lethal lipid peroxides accumulation was considered to be indispensable for ferroptosis [11,28,29,30]. Previous studies also demonstrated that the accumulation of lipid peroxides was associated with the EMP-induced neuronal damages and the subsequent functional impairments [3,4,5,6]. It was proven that electrical pulses could induce the generation of ROS, oxidative damage of unsaturated lipids, and accumulation of lipid peroxides [31]. In particular, the sub-microsecond pulses were responsible for inducing mitochondrial damage and release of ROS to the cytoplasm [32]. The accumulation of ROS could further induce oxidative damage to the cellular membrane system and cause lipid peroxidation [33]. In our in vitro study, the HT22 neurons experienced sub-microsecond electrical pulses with high intensity during the EMP exposure (pulse width = 500 ns and peak electrical field strength = 400 kV/m). After the EMP exposure, the viability decrease, upregulation of PTGS2 mRNA, the accumulation of lipid peroxides (MDA), and downregulation of GPX4 were observed in HT22 neurons. In contrast, after co-treatment with Fer-1, a lipid peroxides scavenger, the putative ferroptosis biomarkers as well as the downregulation of GPX4 were reversed. These findings indicated that EMP caused ferroptosis and inhibition of GPX4 in the hippocampal neurons by enhancing the generation of ROS and the accumulation of lipid peroxides.

The inhibition of the glutathione metabolic pathway was proven to be responsible for initiating ferroptosis. The GSH/GPX4 axis is the essential part of the glutathione metabolic pathway [13,34,35,36]. As a principal antioxidant, GSH is a substrate and cofactor of GPX4. GSH depletion and GPX4 inhibition can cause lipid peroxides accumulation and ultimately trigger ferroptosis. Previous studies reported that EMP exposure can decrease the level of GSH and GPXs in rat hippocampus [3,5,6]. In this study, we found that the level of GSH and the expression of GPX4 decreased significantly after the EMP exposure. More importantly, it was demonstrated that the overexpression of GPX4 in HT22 neurons could effectively suppress the upregulation of lipid peroxides and reverse the ferroptotic neuronal death. This evidence suggested that EMP exposure might cause a vicious cycle of lipid peroxides accumulation and GPX4 downregulation, eventually resulting in ferroptosis.

There is an increasing interest in pharmacologically targeting the nodes of ferroptosis for therapeutic intervention, since ferroptosis has been proven to be one of the core factors in various neurological diseases. Recent studies reported that Fer-1 treatment could effectively protect the brain, kidney, and heart from various diseases by scavenging lipid ROS during ferroptosis [14,36,37,38,39,40]. Moreover, the upregulation of GPX4 via genetic manipulation or treatment with exogenous molecules could also inhibit injuries in neurons, germ cells, and epithelial cells [41,42,43]. In this work, the protective effects of Fer-1 treatment and GPX4 overexpression on EMP-induced ferroptosis were demonstrated, respectively. Both the Fer-1 treatment at various concentrations and GPX4 overexpression could markedly reverse the toxicity in HT22 neurons after the EMP exposure. Taken together, these results suggested that Fer-1 and the GSH/GPX4 axis could be potential candidate intervention nodes for protection against EMP-induced hippocampal neuronal ferroptosis, and they may be further exploited to develop novel therapeutic methods for the corresponding learning and memory deficits.

Previous studies reported that neuronal apoptosis might be one of the significant factors that mediates EMP-induced CNS damage [4,10]. Our results revealed, for the first time, that ferroptosis was a key pathogenic factor in the processes of EMP-induced hippocampal neuronal damage. The findings reported in the previous literature and in the present study suggested that the CNS damages caused by EMP exposure might result from a combination of several forms of neuronal death, including both apoptosis and ferroptosis. However, the interactions between EMP-induced neuronal ferroptosis and other types of neuronal death are still unclear. Further investigations in this field will help us identify the targets of EMP-induced CNS damage and develop corresponding therapeutic strategies.

## 4. Materials and Methods

### 4.1. Animals and Groups

The protocol was approved by the Institutional Animal Care and Use Committee of Beijing Institute of Radiation Medicine (Ethic number: IACUC-DWZX-2020-780). Male specific pathogen-free (SPF) Wistar rats (8 weeks old, 180~220 g) were obtained from the Beijing Vital River Laboratory Animal Technology Co., Ltd. (Beijing, China). The rats were maintained under constant environmental conditions (24 ± 2 °C, 12 h light/dark cycle, 60% relative humidity) with ad libitum access to food and water. The rats were randomly divided into two groups (n = 98): the sham group (sham, n = 46) and the EMP exposure group (EMP, n = 52).

### 4.2. EMP Exposure

**Figure 10:**
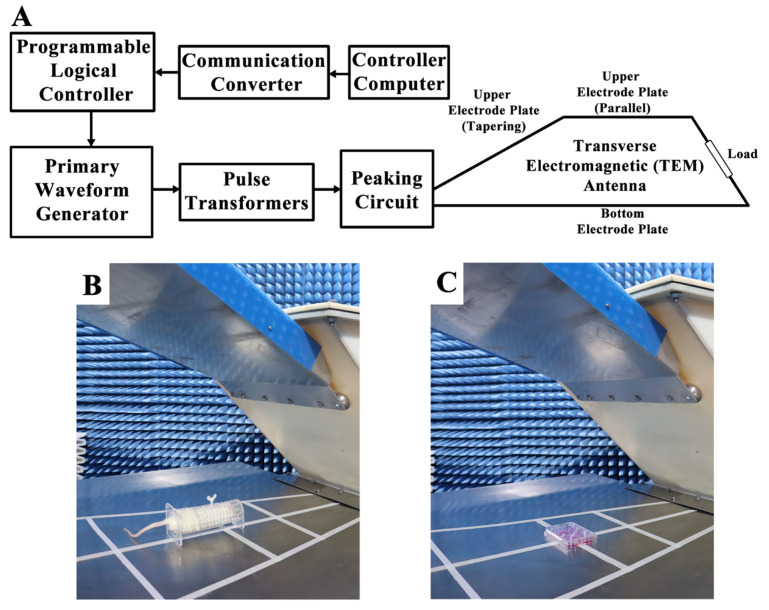
Schematic diagram of the EMP system and exposure scenarios. (**A**) shows the schematic diagram of the EMP system. (**B**,**C**) show the EMP exposure scenarios of rat and cells, respectively.

A vertical polarization bounded wave EMP simulator was implemented in this study. The instrument was developed by the Beijing Institute of Radiation Medicine (Beijing, China) (Figure 10A). Whole bodies of the rats were exposed to EMPs (peak intensity = 400 ± 25kV/m, rise time = 5 ns, pulse width = 500 ns, repetition frequency = 1 Hz, number of pulses = 400). The parameters used in this study were chosen based on the results of the previous literature in order to establish the animal and cell models of EMP-induced neuronal damage [3,4,8,10]. During exposure, the rats were awake and held in specifically designed fixation boxes that were made of plexiglass and free of metal in the exposure chamber (Figure 10B). HT22 cells were also exposed to EMP with the same parameters as those used in rats (Figure 10C). In order to exclude the influences of the environmental factors, rats and cells in the sham group were treated in the same way as the EMP group, except for the exposure. The rectal temperature of the rats and the temperature of the cell culture medium were measured during EMP exposure using a fiber optic thermometer (FOT-m, FISO, Quebec City, QC, Canada) during the animal and cell experiments, respectively.

### 4.3. Morris Water Maze (MWM)

Spatial learning and memory were assessed with the MWM test, as described in the previous literature [44]. The MWM test is a behavioral test that was designed to assess the spatial learning and memory abilities of rodents. The subjects that participate in the MWM test use distal cues to navigate around the perimeter of an open swimming arena to locate a submerged escape platform.

In this study, the MWM test was performed in an open circular pool (diameter = 1.5 m) that was filled with water (temperature = 19–22 °C) and surrounded by curtains. During the MWM test, the computer tracking software Anymaze 6.1 (Stoelting, Wood Dale, IL, USA) was used to record and analyze the behavior of each rat. Before EMP exposure, all rats involved in the experiment were trained using navigation tests for three consecutive days. At 1 d, 2 d, 3 d, 7 d, and 14 d after the EMP exposure, spatial learning was assessed by navigation tests. At 4 d after the EMP exposure, reference memory was assessed by probe trials, as described in the previous literature [44].

In the navigation tests, the circular pool was divided into four equal quadrants: northwest (NW), southeast (SE), northeast (NE), and southwest (SW). A hidden platform was submerged 1–2 cm below the water surface and placed in the middle of the SW quadrant. The rats were placed into the water facing the tank wall. Each session consisted of four trials. In each trial, the rats were released into the water at semirandom starting positions, as proposed by the previous literature [44]. If the rats arrived at the platform within 60 s, the test was terminated, and the average escape latency (AEL) of each rat was recorded. If the rat failed to reach the target platform within 60 s, they were removed from the water, placed onto the target platform, and allowed to remain there for 15 s. The AELs of these rats were recorded as 60 s.

In the probe trials, the target platform was removed. The rats were placed in the water in the NE quadrant facing the tank wall. This placement position, the NE quadrant, was opposite to the target quadrant. All rats were allowed to swim in the pool for 60 s. The percent of time spent in the target quadrant was recorded for each rat.

### 4.4. Sample Collection

After the EMP exposure, the rats were anaesthetized by administration of 1% pentobarbital sodium into the cavum abdominis (50 mg/kg). The rats were then sacrificed. For hematoxylin–eosin (HE) staining, the halves of the brains with the hippocampus were then placed in 10% buffered formalin solution for fixation. For transmission electron microscopy (TEM) analysis, hippocampal tissue (1 mm^3^) was quickly harvested from the CA3 region of the rats. For subsequent molecular analysis, the hippocampus was removed and stored at −80 °C.

### 4.5. HE Staining

The brain tissues collected after the EMP exposure were embedded in paraffin and sliced into 3–5 μm sections in the coronal plane. The sections were deparaffinized and rehydrated using xylene (Sinopharm Chemical Reagent Co., Ltd., Shanghai, China) and alcohol (Sinopharm Chemical Reagent Co., Ltd., Shanghai, China) with different concentrations and then dipped into hematoxylin (ZSGB-BIO, Beijing, China) for 5 min. The sections were then destained in 1% hydrochloric acid for 7 s and redyed in eosin (ZSGB-BIO, Beijing, China) for 2 min. Following dehydration in increasing concentration of alcohols, the sections were put into xylene baths for clearing. Finally, the sections were mounted in neutral balsam (Shanghai Huashen Healing Equipment Co., Ltd., Shanghai, China). After HE staining, the microstructure of the rat hippocampus was observed under optical microscopy (DM6000, Leica, Wetzlar, Germany). The quantitative evaluation of the counts of deeply stained neuron nuclei in rat hippocampus after the EMP exposure was performed using ImageJ 1.48v (National Institutes of Health, Bethesda, MD, USA) to investigate the effects of EMP exposure.

### 4.6. TEM Analysis

The cubes of hippocampal tissues collected after the EMP exposure were placed in 2.5% glutaraldehyde and postfixed with 1% osmium tetroxide. After processing with graded ethyl alcohols, the cubes were embedded in EPON618. The thin sections on copper mesh were stained with heavy metals, uranyl acetate, and lead citrate for contrast. After drying, the hippocampal ultrastructures were observed by TEM (H7650, Hitachi, Tokyo, Japan).

### 4.7. HT22 Cell Cultures

An immortalized mouse hippocampal neuron line (HT22) was obtained from Procell Life Science & Technology (Wuhan, China) and cultured in Dulbecco’s modified Eagle’s medium (Gibco, Carlsbad, CA, USA) supplemented with 10% fetal bovine serum (Gibco, Carlsbad, CA, USA) at 37 °C in a 5% CO_2_ incubator.

### 4.8. Cell Transfection

HT22 cells in the GPX4 overexpression (GPX4-OE) group were transfected with Ubi-GPX4-CBh-gcGFP-IRES-Puro lentiviral vectors (GeneChem, Shanghai, China). Meanwhile, HT22 cells in the negative control (NC) group were transfected with the empty vector (GeneChem, Shanghai, China). For the stable overexpression of the GPX4 gene, the HT22 cells were seeded in 6-well plates and cultured for 24 h. The cells were then transfected with lentiviruses for 10 h and selected in 1.5 µg/mL puromycin (REVG1001, GeneChem, Shanghai, China). Moreover, the stably transfected cells were selected using 0.25 µg/mL puromycin. At last, the efficiency of overexpression was evaluated by Western blot analysis.

### 4.9. Cell Viability Assay

HT22 cells were plated on 96-well plates and cultured with or without 1 μM Fer-1 (Selleck Chemicals, Houston, TX, USA) for 18 h before EMP exposure. Cell viability was evaluated by using a Cell Counting Kit-8 (CCK-8) Colorimetric Assay (CK04, Dojindo, Kumamoto, Japan) according to the manufacturer’s instructions. The average optical densities (ODs) of the plates were measured using a SpectraMax 190-detection microplate reader (Molecular Devices, San Jose, CA, USA) at a wavelength of 450 nm. The average percentage of neuronal viability in each group was calculated by comparison to the sham group.

### 4.10. Cell Death Assay

HT22 cells were plated on 6-well plates and cultured with or without 1 μM Fer-1 (Selleck Chemicals, Houston, TX, USA) for 18 h before EMP exposure. Cell death was evaluated by Calcein-AM/propidium iodide (PI) double staining following the manufacturer’s instructions (C542, Dojindo, Kumamoto, Japan), which was observed under a fluorescence microscopy.

### 4.11. Iron Level Assessment

After the EMP exposure, the Fe^2+^ levels in rat hippocampal tissues were determined using an Iron Assay Kit (MAK025, Sigma-Aldrich, St. Louis, MO, USA) following the manufacturer’s protocols. The average ODs of the samples were measured using a SpectraMax 190-detection microplate reader (Molecular Devices, San Jose, CA, USA) at a wavelength of 593 nm.

HT22 cells were seeded on 8 chambered cover glasses (C8-1-N, Cellvis, Mountain View, CA, USA) and cultured with or without 1 μM Fer-1 (Selleck Chemicals, Houston, TX, USA) for 18 h before EMP exposure. After the EMP exposure, the intracellular Fe^2+^ concentrations of the HT22 cells were measured using a FerroOrange probe (F374, Dojindo, Kumamoto, Japan) following the manufacturer’s protocol. Fluorescence images of HT22 cells were observed using a confocal live-cell time-lapse imaging system (UltraVIEW VOX, PerkinElmer, Waltham, MA, USA).

### 4.12. Lipid Peroxidation Assessment

After the EMP exposure, the levels of lipid peroxidation in rat hippocampal tissues were assessed by using a Lipid Peroxidation malonaldehyde (MDA) Assay Kit (MAK085, Sigma-Aldrich, St. Louis, MO, USA) according to the manufacturer’s instructions. The average ODs of the samples were measured using a SpectraMax 190-detection microplate reader (Molecular Devices, San Jose, CA, USA) at a wavelength of 532 nm.

HT22 cells were seeded in 6-well plates and cultured with or without 1 μM Fer-1 (Selleck Chemicals, Houston, TX, USA) for 18 h before EMP exposure. After the EMP exposure, the levels of lipid peroxidation in HT22 cells were measured using the same protocol as that implemented in the corresponding hippocampus experiments.

### 4.13. GSH Assessment

HT22 cells were seeded on 6-well plates and cultured. After the EMP exposure, the levels of reduced GSH in the HT22 cells were measured using an assay kit (Nanjing Jiancheng Bioengineering Institute, Nanjing, China).

### 4.14. Real-Time PCR Analysis

After the EMP exposure, total RNA was harvested from rat hippocampal tissues using TRIzol (Ambion, Life Technologies, Carlsbad, CA, USA) and reverse transcribed into complement DNA using PrimeScript RT Master Mix (RR036A, Takara, Shiga, Japan). Real-time PCR (RT-PCR) was carried out using TB Green Premix Ex Taq II (RR820A, Takara, Shiga, Japan) and then performed on an Applied Biosystems 7300 Real-Time PCR System (Thermo Fisher Scientific, Carlsbad, CA, USA). All the samples were assayed in triplicate, and differences in the relative mRNA expression of the ferroptosis-related gene PTGS2 were determined using the delta-delta cycle threshold (ddCt) method, with glyceraldehyde 3-phosphate dehydrogenase (GAPDH) as the internal reference control. For the RT-PCR analysis of rat hippocampal tissues, the following primers were used: PTGS2, forward 5′-TGAACACGGACTTGCTCACTTTG-3′ and reverse 5′-AGGCCTTTGCCACTGCTTGTA-3′; and GAPDH, forward 5′-GGCACAGTCAAGGCTGAGAATG-3′ and reverse 5′-ATGGTGGTGAAGACGCCAGTA-3′.

HT22 cells were seeded in 6-well plates and cultured with or without 1 μM Fer-1 (Selleck Chemicals, Houston, TX, USA) for 18 h before EMP exposure. After the EMP exposure, RT-PCR analysis of HT22 cells was performed using the same procedures as those used for the hippocampus tissues. For the RT-PCR analysis of mouse HT22 cell transcripts, the following primers were used: PTGS2, forward 5′-CTGGAACATGGACTCACTCAGTTTG-3′ and reverse 5′-AGGCCTTTGCCACTGCTTGTA-3′; solute carrier family 7 member 11 (SLC7A11), forward 5′-TGGGCTACGTACTGACAAACGTG-3′ and reverse 5′-GCAACAAAGATCGGGACTGCTAA-3′; nuclear factor E2-related factor 2 (NRF2), forward 5′-AGTGACTCGGAAATGGAGGAG-3′ and reverse 5′-TGTGCTGGCTGTGCTTTAGG-3′; and GAPDH, forward 5′-TGTGTCCGTCGTGGATCTGA-3′ and reverse 5′-TTGCTGTTGAAGT CGCAGGAG-3′.

### 4.15. Western Blotting

After the EMP exposure, total protein was extracted from rat hippocampal tissues with a RIPA lysis buffer (P0013B, Beyotime Biotechnology, Shanghai, China) supplemented with a protease inhibitor cocktail (B14001, Bimake, Houston, TX, USA). The protein concentration was detected by the Pierce BCA Protein Assay Kit (23225, Thermo Fisher Scientific, Carlsbad, CA, USA). Lysates were run using a sample preparation kit (Protein Simple) on the automated capillary Western blot system, JESS System (Protein Simple). Then, the levels of the ferroptosis-related antioxidant GPX4 were analyzed. GAPDH (Abcam, Cambridge, UK) was used as the loading control.

HT22 cells were seeded in 6-well plates and cultured with or without 1 μM Fer-1 (Selleck Chemicals, Houston, TX, USA) for 18 h before EMP exposure. After the EMP exposure, the levels of GPX4, SLC7A11, and NRF2 in the HT22 cells were analyzed by Western blotting according to standard protocols using primary antibodies specific for GPX4 (ab125066, Abcam, Cambridge, UK), SLC7A11 (ab175186, Abcam, Cambridge, UK), and NRF2 (ab92946, Abcam, Cambridge, UK). GAPDH (Abcam, Cambridge, UK) was used as the loading control. Horseradish peroxidase (HRP)-conjugated anti-mouse and anti-rabbit secondary antibodies (ab6728; ab6721, Abcam, Cambridge, UK) were used, and the signals were detected using a Pierce ECL Western blotting detection system (CLINX, Shanghai, China). For protein quantification, the densities of the Western blot bands were measured by the ImageJ 1.48v (National Institutes of Health, Bethesda, MD, USA).

To evaluate the efficiency of GPX4 overexpression in GPX4-OE HT22 neurons, cells were collected and lysed with a RIPA lysis buffer (P0013B, Beyotime Biotechnology, Shanghai, China) supplemented with a protease inhibitor cocktail (B14001, Bimake, Houston, TX, USA). The levels of the GPX4 protein were measured using the same procedures as those implemented in the corresponding hippocampal tissue experiments.

### 4.16. Statistical Analysis

All the data are presented as the mean ± SEM for each group. Statistical analyses were performed using IBM SPSS Statistics Version 19 (IBM, Chicago, IL, USA). The comparisons between only two groups were performed with two-tailed unpaired Student’s t-test or paired-samples *t*-test. A one-way ANOVA followed by LSD post hoc test was performed for comparisons involving more than two groups. Cell viability, Fe^2+^ levels, MDA levels, GSH levels, and GPX4, PTGS2, SLC7A11, and NRF2 expression data were normalized to those of the sham group. Statistical significance for all measurements was set to *p* < 0.05 (95% confidence interval).

## 5. Conclusions

**Figure 11:**
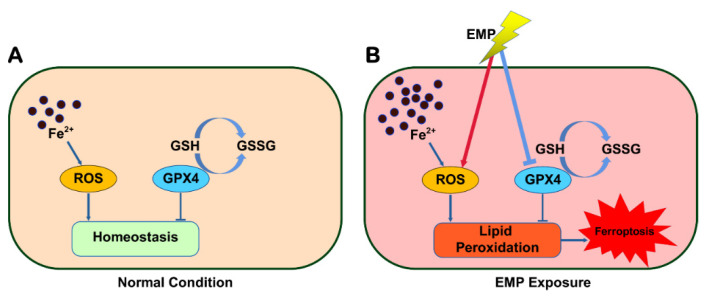
Schematic representation of the potential mechanism involved in EMP-induced hippocampal neuronal ferroptosis.

In this work, for the first time, we revealed that ferroptosis played a significant role in EMP-induced hippocampal neuronal damage. EMP induced ferroptosis in hippocampal neurons via a vicious cycle of lipid peroxides accumulation and GPX4 downregulation (Figure 11). Fortunately, inhibiting ferroptosis may represent a feasible therapeutic approach for managing EMP-induced hippocampal neuronal damage. This work could not only shed light on the underlying mechanism of EMP-induced hippocampal neuronal damage but also help us develop the corresponding therapeutic strategies.

## Data Availability

Not applicable.

## Abbreviations

AEL: average escaping latency; Calcein-AM, Calcein acetoxymethyl ester; CNS, central nervous system; ddCt, delta-delta cycle threshold; DG, dentate gyrus; EMP, electromagnetic pulse; Fe^2+^, ferrous ion; Fer-1, ferrostatin-1; GAPDH, glyceraldehyde 3-phosphate dehydrogenase; gcGFP, gc green fluorescent protein; GPX4, glutathione peroxidase 4; GPX4-OE, glutathione peroxidase 4 overexpression; GSH, glutathione; GPXs, glutathione peroxidases; HE, hematoxylin–eosin; HRP, horseradish peroxidase; LOOHs, lipid hydroperoxides; MDA, malonaldehyde; MWM, Morris water maze; NC, negative control; NE, northeast; NRF2, nuclear factor E2-related factor 2; NW, northwest; ODs, optical densities; PI, propidium iodide; PLOOH, phospholipid hydroperoxides; PTGS2, prostaglandin-endoperoxide synthase 2; RCD, regulated cell death; RT-PCR, real-time PCR; SE, southeast; SLC7A11, solute carrier family 7 member 11; SPF, specified pathogen-free; SW, southwest; TEM, transmission electron microscopy; WHO, World Health Organization.
